# Supplementary material for: AXIS: a Lab-in-the-Loop machine learning approach for automated detection of macromolecular crystals
Source: IUCrJ. 2026 Mar 19;13(Pt 3):235–48. doi: 10.1107/S2052252526001399 (PMC13134493; doi:10.1107/S2052252526001399)

IUCrJ

Volume 13 (2026)

Supporting information for article:

AXIS: a Lab-in-the-Loop machine learning approach for automated detection of macromolecular crystals

Aurelien Personnaz, Sihyun Sung, Raphael Bourgeas, Sruthi Unni, Florine Dupeux, Bukunmi Adediran, Rosicler Barbosa, Anne-Sophie Humm, Euan Colaco-Osorio and José Antonio Márquez

Figure S1 Confusion matrices of the different AXIS classifiers with the CRIMS-test dataset

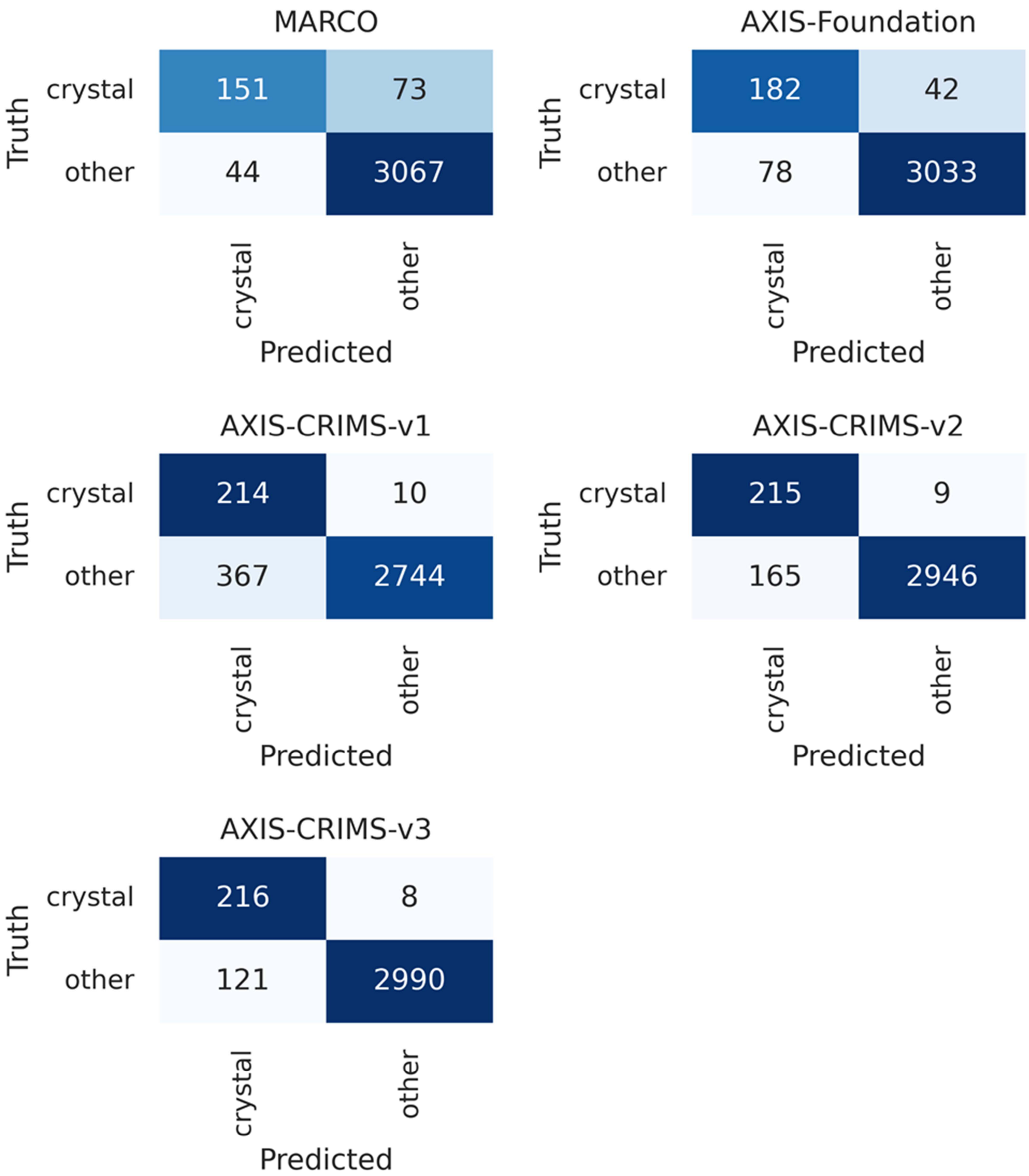

Supplement: Supplementary file 1 [file m-13-00235-sup1.pdf]
